# Utility of microbial cell-free DNA sequencing in the diagnosis of mycobacterial infections in a quaternary care center by Centeno et al.

**DOI:** 10.1017/ash.2025.10209

**Published:** 2026-06-01

**Authors:** Frederick S. Nolte

**Affiliations:** Medical Affairs, Karius, Inc., Smyrna, USA

We read with great interest the article by Centeno *et al.* evaluating the use of microbial cell-free DNA (mcfDNA) sequencing in the diagnosis of mycobacterial infections.^1^ We commend the authors for highlighting the diagnostic and clinical utility of mcfDNA sequencing in challenging infectious disease cases. However, we respectfully wish to correct an important factual error regarding the reported cost of the Karius Spectrum test. On page 3, the authors state: *“Additionally, mcfDNA NGS remains costly at $5,494 compared to $231 for MTB PCR and $4,325 for daily ICU charges.”* This figure does not reflect the current price of the test. The actual list price is $2,200, billed directly to the hospital. We presume the higher figure reported represents the hospital’s charge after markup.

Hospital markups for laboratory tests can be substantial, often three to five times the actual cost, or more.^2^ These markups vary widely by institution, leading to significant differences in what patients are billed for the same test, and influencing both out-of-pocket costs and overall healthcare spending.

Price transparency and accuracy are essential for diagnostic stewardship, cost-effectiveness analyses and healthcare economics and outcomes research. We urge researchers to use the true cost of the test when conducting such analyses to ensure accuracy and fairness.
